# Technical feasibility of multimodal imaging in neonatal hypoxic-ischemic encephalopathy from an ovine model to a human case series

**DOI:** 10.3389/fped.2023.1072663

**Published:** 2023-06-22

**Authors:** Brian Hagan, Radhika Mujumdar, Jagdish P. Sahoo, Abhijit Das, Anirban Dutta

**Affiliations:** ^1^ School of Engineering, University of Lincoln, Lincoln, United Kingdom; ^2^ Department of Neonatology, IMS & SUM Hospital, Bhubaneswar, India; ^3^ Department of Neurology, The Lancashire Teaching Hospitals NHS Foundation Trust, Preston, United Kingdom

**Keywords:** hypoxic-ischemic encephalopathy, electroencephalogram, near-infrared spectroscopy, neurovascular coupling, experimental modal analysis

## Abstract

Hypoxic-ischemic encephalopathy (HIE) secondary to perinatal asphyxia occurs when the brain does not receive enough oxygen and blood. A surrogate marker for “intact survival” is necessary for the successful management of HIE. The severity of HIE can be classified based on clinical presentation, including the presence of seizures, using a clinical classification scale called Sarnat staging; however, Sarnat staging is subjective, and the score changes over time. Furthermore, seizures are difficult to detect clinically and are associated with a poor prognosis. Therefore, a tool for continuous monitoring on the cot side is necessary, for example, an electroencephalogram (EEG) that noninvasively measures the electrical activity of the brain from the scalp. Then, multimodal brain imaging, when combined with functional near-infrared spectroscopy (fNIRS), can capture the neurovascular coupling (NVC) status. In this study, we first tested the feasibility of a low-cost EEG-fNIRS imaging system to differentiate between normal, hypoxic, and ictal states in a perinatal ovine hypoxia model. Here, the objective was to evaluate a portable cot-side device and perform autoregressive with extra input (ARX) modeling to capture the perinatal ovine brain states during a simulated HIE injury. So, ARX parameters were tested with a linear classifier using a single differential channel EEG, with varying states of tissue oxygenation detected using fNIRS, to label simulated HIE states in the ovine model. Then, we showed the technical feasibility of the low-cost EEG-fNIRS device and ARX modeling with support vector machine classification for a human HIE case series with and without sepsis. The classifier trained with the ovine hypoxia data labeled ten severe HIE human cases (with and without sepsis) as the “hypoxia” group and the four moderate HIE human cases as the “control” group. Furthermore, we showed the feasibility of experimental modal analysis (EMA) based on the ARX model to investigate the NVC dynamics using EEG-fNIRS joint-imaging data that differentiated six severe HIE human cases without sepsis from four severe HIE human cases with sepsis. In conclusion, our study showed the technical feasibility of EEG-fNIRS imaging, ARX modeling of NVC for HIE classification, and EMA that may provide a biomarker of sepsis effects on the NVC in HIE.

## 1. Introduction

Hypoxic-ischemic encephalopathy (HIE) is one of the most common causes of neonatal death worldwide, accounting for approximately 23% of all neonatal deaths (1). Worldwide, it is estimated to account for more than one million deaths annually. HIE also accounts for considerably higher numbers of chronic neurological deficits that create an economic burden, more so in developing countries. Despite having such an immense societal impact, an adequate rapid diagnostic method for HIE is lacking (2). In HIE, there is a prolonged lack of oxygen entering the brain, which causes serious neuronal damage within a very short window of time, approximately 2–3 min if complete lack of oxygen, and will lead to a cessation of any neuronal activity shortly thereafter. Due to the direct effect of HIE on the neuronal state, current monitoring and outcome prediction are predominantly based on the electroencephalogram (EEG), which measures neuronal activity in the cerebral cortex. Amplitude-integrated EEG (aEEG) is an effective prognostic method for long-term neurologic deficits induced by HIE with a 90% classification accuracy at 6 h after injury in both positive and negative predictions. Here, positive predictions dictate that a subject will have significant deficits caused by the hypoxic event and negative predictions characterize recovery of normal neuronal function after the injury. In most studies, the least time to obtain an accepted and accurate prediction of extended deficits was found to be around 6 h and the lowest acceptable was 3 h, where the positive prediction scores were below 80%, and prior to that time window, the method did not provide any consistent predictive value (3, 4). Other alternative methods for prognosis in HIE such as magnetic resonance imaging (MRI) have fallen out of favor as they lack prognostic ability or speed in the early stage of brain injury. For example, T1- and T2-weighted MRI takes approximately 1 week for an accurate prognosis resulting from brain swelling from the injury. Also, there is a lack of MRI facilities in resource-poor settings and it is often cost-prohibitive. According to a meta-analysis by van Laerhoven (5), the diagnosis is at best on par with the 6-h aEEG with a positive predictive score of 83% and a negative score of 90%. Then, Chalak et al. (6) presented a neurovascular coupling (NVC)-based approach in HIE using multimodal imaging with aEEG combined with functional near-infrared spectroscopy (fNIRS) and wavelet coherence analysis. Here, the challenge remains in the continuous monitoring of NVC, where Sood et al. (7) presented a Kalman filter-based method that allowed online autoregressive with extra input (ARX) parameter estimation using time-varying signals and could capture transients in the coupling relationship between EEG and fNIRS signals. Then, the availability of low-cost portable brain imaging devices, e.g., OpenBCI (https://openbci.com/) and M3BA (8), can be leveraged for clinical translation of continuous cot-side brain monitoring in limited resource settings that can potentially help for better management of neonates with perinatal asphyxia and improve the long-term neurodevelopmental outcome. In the current study, the overarching objective was to test the feasibility of a low-cost multimodal brain imaging device (8) and an ARX-based support vector machine (SVM) classifier for point-of-care HIE monitoring in limited resource settings.

Continuous monitoring of neurovascular coupling may be superior to clinical scores for HIE classification (9). The hypoxic state can be rapidly detrimental for the brain neurons due to a large amount of oxygen needed in continuous supply (∼10 ml/100 g tissue/min) and its low reserve, leading to large changes in neuronal firing during oxygen deficits that can affect the EEG power spectrum. Indeed, hypoxia effects on the EEG power spectrum have been extensively studied in both humans and animal models (10, 11). The spectral density, more commonly referred to as the power spectrum of the signal, makes the EEG signal easier to analyze based on rhythms that can be monitored over time as a spectrogram. The effect of hypoxia on the power spectrum has been studied using animal models (12); for example, Goel et al. (13) in an animal model of a neonatal piglet showed results from hypobaric hypoxia that was induced for 30 min using 10% oxygen concentration in air. Then, the airway was occluded for   min, during which the piglet's neural firing ceased, and the piglet was resuscitated afterward. Throughout the protocol, the EEG was monitored while the piglet was anesthetized. The power spectrum was calculated at the end of both segments, airway occlusion and resuscitation, and one remarkable feature was spectral dispersion, where the low-frequency alpha and theta firings were most affected by hypoxia; also, there was a degree of disproportionality in the recovery of power of the three dominant frequency bands (1.0–5.5, 9.0–14.0, and 18.0–21.0 Hz) relative to their mean recovered power. Time domain features, such as Hjorth parameters, have also been used. The Hjorth parameters are simple statistical calculations on the EEG signal, with the first parameter known as the activity of the signal, which is the variance of the amplitude for a window of the signal in time, and the second Hjorth parameter known as the mobility of the signal. Mobility is defined as the square root of the ratio of the first parameter of the rate of change of the signal, divided by the actual first parameter of the signal, or the rate of change of the activity divided by the activity of the signal. The last of the Hjorth parameters is known as the complexity of the signal, which is the second derivative of the activity divided by the first derivative of the activity. Each of these Hjorth parameters changed during HIE and was found useful, especially in the classification of early partial seizure onset (14). Then, aEEG is a major clinical tool for the long-term prognosis of HIE; however, it uses 10-min windows for calculation and needs at least 6 h of data for accurate prognosis. Here, aEEG on its own needs prolonged data acquisition for an accurate prognosis that may outrun the early treatment window for HIE. Also, aEEG can be processed using Washington University-Neonatal EEG Analysis Toolbox (WU-NEAT) to estimate NVC in conjunction with fNIRS (9), which can be used for HIE classification (6). Another time series analysis is autoregressive (AR) modeling, which takes a segment of data and fits it to the current data point in a linear combination of previous data points multiplied by parameters that have a fixed value throughout the segment. The AR model requires matrix calculations to acquire these parameter values and requires validation to ensure that it is adequately capturing the EEG signal properties and not the noise especially when detecting seizure activity (15). If the AR model adequately fits the data, the power spectrum trends are captured in the transfer function output of the system model and can be reconstructed. Then, operational modal analysis using AR with the eXogenous input (ARX) model can provide mechanistic insights from the NVC system model with the simultaneously acquired EEG-fNIRS data. We have extended published algorithms for online multimodal brain imaging using EEG and fNIRS in our prior work (7).

The current study is motivated by recent findings on the role of NVC in the prediction of brain abnormalities in neonatal encephalopathy (9). Das et al. (9) found NVC to be a promising biomarker in neonatal HIE that was superior to the total Sarnat score (16) for the prediction of abnormal brain MRI in the later stages. In estimating coherence, stationarity and ergodicity of the signal are assumed, which needs preprocessing of the raw EEG data to remove trends and low-frequency variations. Then, the modeling accuracy becomes more challenging when the spectra contain sharp peaks, e.g., during rhythmic activity (17). Therefore, an ictal classifier based on EEG spectral features was developed using the Children's Hospital of Boston and the Massachusetts Institute of Technology (CHB-MIT) dataset (18) to separately label seizure activity (19). Then, a SVM was used with the AR parameters to classify EEG (20) into various experimentally induced states in an ovine model of perinatal asphyxial arrest (21). Here, we applied AR modeling and assumed AR parameters being constant throughout the selected window size (22). Ahmed et al. (20) have used a multiclass SVM classifier for the best estimation of an outcome based on a commonly used clinical grade of one to four: a grade of one being non- to mild abnormalities, two being moderate, three being major EEG depression, and four being a severe EEG discontinuity. Their classifier overall had an 87% accuracy in classifying the recovery grade of newborns from HIE and was found to be one of the most effective such classifiers, while others were as accurate as 77% (23). Here, we also performed ARX modeling using EEG-fNIRS data from the ovine model of perinatal asphyxial arrest (21). The objective was to test the feasibility of a low-cost EEG-fNIRS device and the ARX-based linear classifier to label simulated HIE states in a perinatal ovine hypoxia model. Then, we applied the ARX-based linear classifier trained with perinatal ovine hypoxia model data to a human case series on perinatal HIE with and without sepsis. We also investigated experimental modal analysis (EMA) of the NVC system model that provided mechanistic insights from simultaneously acquired EEG-fNIRS data. Here, the ARX model allowed the estimation of the modal parameters and frequency response functions (FRFs) of the NVC system. Then, the FRFs of the EEG power as input and the hemodynamic (fNIRS) changes as output were used for the EMA of the NVC system dynamics for the mechanistic insights into the HIE (with vs. without sepsis).

## 2. Materials and methods

### 2.1. Animal model and data processing

**Figure 1:**
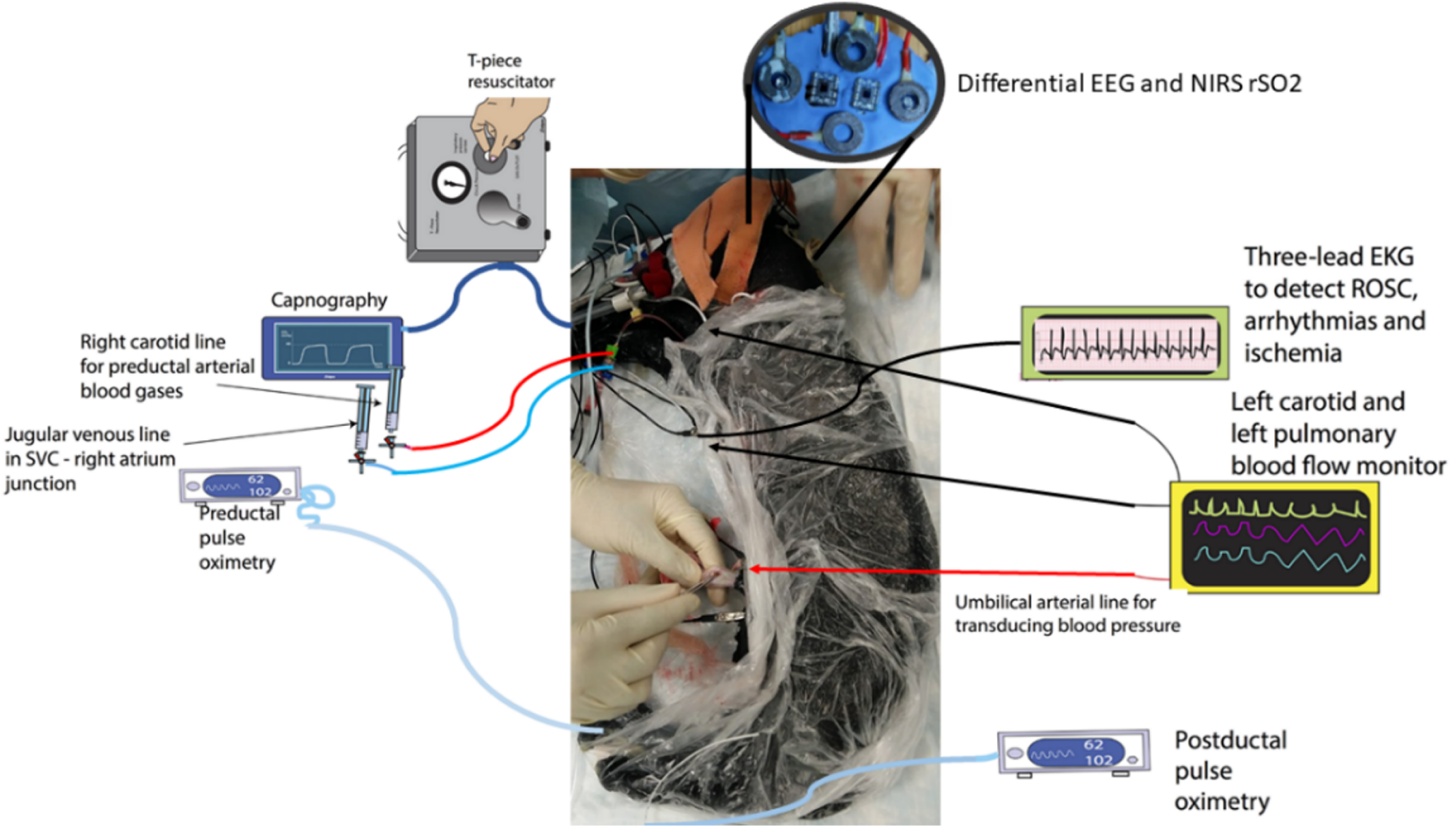
Differential EEG and the fNIRS sensors were added to the perinatal asphyxiated lamb model experiments (26). The figure was adapted from Figure 1 of Vali et al. (25). EEG, electroencephalogram; fNIRS, functional near-infrared spectroscopy.

The preparation of ovine subjects was carried out in accordance with the Institutional Animal Care and Use Committee at the State University of New York at Buffalo, United States (24–26). Term (140–147 days) pregnant ewes were obtained from New Pasteur Family Farms (Attica, NY, United States). After an overnight fast, the pregnant ewe was anesthetized with intravenous diazepam and ketamine. The ewe was continuously monitored using a pulse oximeter and an end-tidal carbon dioxide monitor. The ewe was intubated with a 10-mm cuffed endotracheal tube and ventilated with 21% oxygen and 2%–3% isoflurane at a breathing rate of 16 breaths per minute. The perinatal ovines were delivered by a cesarean section and partially exteriorized and intubated. Once the delivery process was completed, excess fluid that remained in the lungs of the newborn was removed via passive measures, by tilting the head back and forth for simulating the process by which fluid is removed during birth. Once the excess liquid was removed, the airway was occluded to prevent gas exchange. The catheters were then placed in the jugular vein and right carotid artery to sample blood and administer any necessary medication. A 2-mm flow probe (Transonic Systems Inc., Ithaca, NY, United States) was placed around the left carotid artery and a 4-mm flow probe was placed in the left pulmonary artery. The electrocardiogram electrodes were then placed in the right and left axilla and right inguinal area, a standard three-lead setup. The ECG100C (Biopac, Inc.) was used with Acknowledge software to record data from leads I, II, and III of the ECG. The saturation of preductal arterial oxygenated hemoglobin was monitored by a pulse oximeter placed on the right forelimb of the neonate. Low-cost wireless EEG-fNIRS (750 nm and 850 nm) sensors (OEM from Technische Universität Berlin) (8) were placed on the forehead for continuous measurement at 500 Hz for EEG and 10 Hz for fNIRS (see Figure 1). Our low-cost wireless EEG-fNIRS (750 nm and 850 nm) sensors (Bionics Institute, Australia) were validated using off-the-shelf EEG (Biopac Inc., United States) and fNIRS (Nonin Medical, United States) sensor data from the established perinatal asphyxiated lamb model experiments; see the experimental protocol by Vali et al. (26).

**Figure 2:**
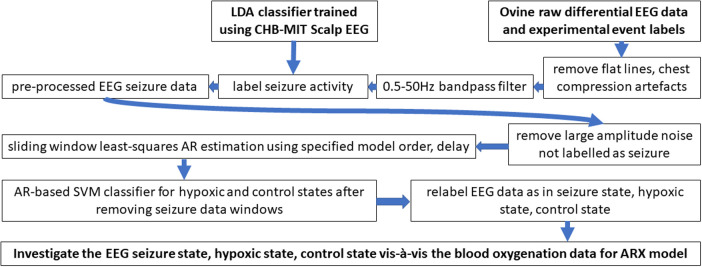
Algorithmic workflow for processing perinatal ovine EEG data. EEG, electroencephalogram.

Following instrumentation, the umbilical cord was occluded until asystole, which is defined as the complete lack of carotid artery flow, arterial blood pressure, and heart rate. The lamb remained in the asystole for 5 min, and then resuscitation was started. Positive pressure ventilation (PPV) via an endotracheal tube was provided with 20% oxygen and was performed using a T piece at a rate of 40 breaths per minute (25). After 1 min of ventilation, chest compressions (CCs) were initiated and coordinated with CCs in the ratio of 3:1 (3 CC: 1 PPV). After 5 min of resuscitation, if the lambs did not have a spontaneous return of circulation (ROSC—defined as heart rate >60/m with systolic blood pressures >30 mmHg), medications (epinephrine or vasopressin) were administered through an umbilical venous catheter. Blood gases were obtained at intervals, and the lambs were ventilated after ROSC for 2–3 h. If the lambs had ROSC, resuscitation was stopped at 20 min. Data from five ovine subjects were analyzed in this study with a gestational time of 139–142 days, as shown in Table 1. The data analysis followed five major portions: raw data extraction, preprocessing, autoregressive modeling, classification, and validation. The workflow presented in Figure 2 was used to obtain results from the EEG and fNIRS systems starting with the extraction of raw data.

**Table 1:** Five full-term ovine subjects used in the animal study.

Subjects	Gestational time (days)	Weight (lbs)	Sex
11/16-1	142	5.5	Male
12/13	141	3.05	Female
3/26	139	4	Male
11/16-2	142	5.5	Male
11/15	141	4.4	Male

#### 2.1.1. Major events were labeled offline as follows

First, we start the EEG or initiate our experiment; then, neonate delivery was performed, followed by the asphyxiation of the subject. Here, from the beginning of EEG to the start of asphyxiation, the data were labeled as control or normal. The next major event was the point of no cardiovascular function, known as asystole. Data between the time of neonate delivery and asystole were labeled as ischemic data. The next event was the start of resuscitation. The data between the asystole and the start of resuscitation were labeled the asystole segment. The two portions consisting of ischemic and asystole data segments were combined into a more generalized hypoxia phase for our AR-SVM classifier testing. The last event was the recovery of spontaneous circulation or ROSC. So, the data segment between the start of resuscitation and the ROSC was labeled as the chest compression segment, and then, the last segment was the recovery segment in the case of ROSC of the subject.

##### 2.1.1.1. Data preprocessing—removal of chest compression

As EEG is affected by the movement artifacts due to chest compressions, the data segment was completely removed during preprocessing.

##### 2.1.1.2. Data preprocessing—removal of flat lines

The next stage of preprocessing was to remove sections of the data where electrode contact was lost or obstructed, which can appear as a flat-line artifact in the EEG. The EEG flat lines were removed by measuring the standard deviation of the signal in the sliding window after visual confirmation.

##### 2.1.1.3. Data preprocessing—correcting for baseline drift

The removal of the baseline drift is the next step in the preprocessing pipeline. EEG is considered a zero mean signal; therefore, if the signal mean is not zero over time, then it was considered a baseline drift artifact. In the case of a baseline drift artifact, the EEG data were adjusted back to a zero mean using the “detrend” function in MATLAB (MathWorks, Inc.).

##### 2.1.1.4. Data preprocessing—bandpass filtering of data

A bandpass filter was designed with cutoff frequencies set to 0.5–50 Hz [neonates rarely have high gamma activity (27)], thereby removing both low-frequency artifacts (common causes of nonstationarity) and high-frequency noise. We applied a fifth-order filter that provided a stable filter for our specific cutoff frequencies using the “butter” function in MATLAB (MathWorks, Inc.).

##### 2.1.1.5. Data preprocessing—LDA binary classifier for seizure

The next stage of the preprocessing pipeline was to label ictal activity using a sliding window of the data (22) as seizure activity occurred frequently, and we aimed at hypoxia classification using background (nonseizure) EEG activity (28). Linear discriminant classifier (LDA) classifier training used the CHB-MIT dataset (18). The CHB-MIT scalp EEG dataset contains 22 subjects from children who have been removed from antiepileptic medication and suffered seizures. The sampling rate for all data was 256 Hz, and the international 10–20 standard montage was used for recording. A physician trained in EEG-based seizure detection manually labeled the occurrence of ictal activity in the CHB-MIT dataset. To maximize the scalability of the LDA classifier from the CHB-MIT dataset to our EEG measurement, only one channel of differential data was chosen for training the classifier, as described next.

The differential EEG channel used in our perinatal ovine study was comparable to the Fz-Cz electrode pair from the human 10–20 labels. Therefore, Fz-Cz electrode data were processed for differential EEG like our perinatal ovine data but we did not use their (19) SVM classifier that had several hyperparameters. We used a simpler LDA that was trained using the labeled CHB-MIT dataset [using 3.4-s sliding window that was longer than 2 s used in their SVM classifier (19)]. Our chosen features were alpha and low gamma band power (29). Here, LDA is a binary classifier that generates a linear decision plane to maximize the accuracy of binary classification. To avoid overfitting, a fivefold cross-validation was used, which divided the EEG data into five segments and used four-fifth of the data to train the classifier and one-fifth to test and performed this processing five different times, so all the EEG data can be used for both testing and training. This method is widely used in machine learning to avoid generating overfit classifiers. Once the LDA classifier was trained by the labeled CHB-MIT dataset, the Fz-Cz EEG ovine data in a 2-s sliding window was run through the binary classifier, and the windows with seizure were labeled.

##### 2.1.1.6. Data preprocessing—removal of large-amplitude data segments

The last in the preprocessing was to remove data segments containing large activity (and not labeled as a seizure); this was done by finding the overall standard deviation of the EEG data and removing data segments that had a mean larger than two standard deviations of the whole EEG data.

##### 2.1.1.7. Autoregressive (AR) modeling of the EEG data

The AR model is a linear model that fits the current output using a defined number of previous outputs, multiplied by the same number of coefficients, known as AR parameters. The AR parameters are optimized based on the linear algebra principle of least-squared estimate for the best fit, where the AR model yields higher resolution for spectral analysis than nonparametric approaches when the signal length is short. If an accurate AR model is constructed, then the spectral analysis of the signal can be solely described and reconstructed from AR parameters. AR model delay was included in our model, and the delay calculation was done using autocorrelation, which is a measure of mutual information shared between the signal and a time-shifted version of itself. The analysis of the data autocorrelation was performed for every possible positive delay. The maximum value denotes the time point when there is the most shared information. The delay was calculated for each ovine subject separately from the control phase of the EEG data. The AR model order is the other property of the model that must be optimized, which was done to make sure that the system is being accurately modeled while also being the least computationally expensive, e.g., using Akaike's information criterion (AIC). Prior work (13) also found an optimal AR model order of six for a similar kind of EEG data. After AR modeling the EEG data, these AR parameters were plotted in 3D to visualize clusters from the nonictal background EEG activity. The ictal activity was labeled using a first-level LDA classifier—see section *Preprocessing—seizure binary classifier*. Then, AR parameters were used as features to visualize the clusters for all the three different experimental conditions in the 3D AR feature space; seizure state, hypoxia state, and normal/control state.

##### 2.1.1.8. SVM classification using EEG AR parameters

Separation into the three experimentally induced states, normal/control, hypoxia, and seizure, was done using two different linear classifiers in the hierarchy. The first of the two linear classifiers in the hierarchy was the LDA seizure classifier that was trained using the CHB-MIT dataset—see section *Preprocessing—seizure binary classifier*. This binary classifier was applied to identify the data segments that contained ictal activity, which were removed before training and testing the second-level SVM classifier using the background EEG (3.4-s sliding window). The second-level SVM classifier was used to classify the AR parameters from the seizure-free (background) EEG data segments into the hypoxia state and the control (normal) state. Here, AR parameters were used as features (see Supplementary material Figures S1–S3), and the response variable used for training and validation were the event markers from the animal experiment. The SVM classifier was chosen for a more generalizable decision plane since this SVM classifier that was trained using perinatal ovine data was then applied to human perinatal case series. To avoid overfitting the SVM classifier, a fivefold cross-validation was used.

##### 2.1.1.9. SVM classification using EEG-fNIRS ARX parameters

We applied the ARX model to the EEG-fNIRS data (here, a 60-s sliding window was used due to a slower fNIRS signal) for the second-level SVM classifier using seizure-free data segments for labeling hypoxia and control (normal) states. We used the basic nirs-toolbox (30) script in MATLAB (MathWorks, Inc.) to process the fNIRS data (750 and 850 nm). Specifically, we used the following modules with default parameters: nirs.modules.OpticalDensity, nirs.modules.BeerLambertLaw, and nirs.modules.AR_IRLS. We used the AR-IRLS model (31) that employed both prewhitening and robust regression to remove noise from the data. The ARX model order of six from AIC was comparable to our previous work (7) that used the fNIRS oxyhemoglobin signal in the low-frequency (0.1 Hz) range as the output and the transformed EEG band power as the input (7). In this study, we used an EEG frequency band of 1.0–21.0 Hz due to the dominant frequencies found in a related prior work (13). Then, the ARX parameters (“arx” in MATLAB) were used as features in the SVM classifier, and the response variable used was the event markers from our animal experiment. To avoid overfitting the classifier, a fivefold cross-validation was used.

##### 2.1.1.10. Hierarchical classifier outcome vis-à-vis carotid flow

After the hierarchical classifier was found from the perinatal ovine data, the classifier outcome was compared with the carotid blood flow data. Here, the objective was to compare the changes in the carotid flow rate (irrespective of the manually placed event boundaries) when the ovine subject physiologically entered the global hypoxia stage.

### 2.2. Human data acquisition and feasibility testing

The human perinatal study was conducted based on convenience sampling at the Department of Neonatology and approved by the Institutional Review Board (IRB) of the IMS & SUM Hospital, Bhubaneswar, India. The study objectives were to test the feasibility of the EEG-fNIRS joint imaging for the ARX-based SVM classifier that was trained with the perinatal ovine data to detect the severity of human perinatal HIE. The study was a prospective observational study. Ten newborns with moderate to severe HIE and four severe HIE cases with sepsis were recruited for the feasibility study. Sepsis screening was performed according to the clinical guidelines at the IMS & SUM Hospital, Bhubaneswar, India. Specifically, sepsis was suspected when there was a history of lethargy, poor feeding, fever, hypothermia, or temperature instability, abdominal distension, feeding intolerance, and tachypnea. The suspicion was corroborated with a positive sepsis screen (total leukocyte count < 5,000/cmm or absolute neutrophil count < 1,800/cmm, micro-ESR > 15 mm in the first hour, immature-to-total neutrophil ratio > 0.2, CRP > 10 mg/dl, any two of the four positive parameters meant sepsis screen positive). Sepsis was also confirmed if the blood culture was positive. Here, the physical and neurological examination was performed by neonatologists trained with Sarnat and Sarnat scoring criteria (32).

The inclusion and exclusion criteria were the following:

• Inclusion criteria: Neonates with gestation >35 weeks and >1,800 g admitted to the neonatal intensive care unit (NICU) for the treatment of perinatal asphyxia.
• Exclusion criteria: Premature babies <35 weeks, babies with multiple congenital anomalies, and not giving consent for inclusion in the study.

**Figure 3:**
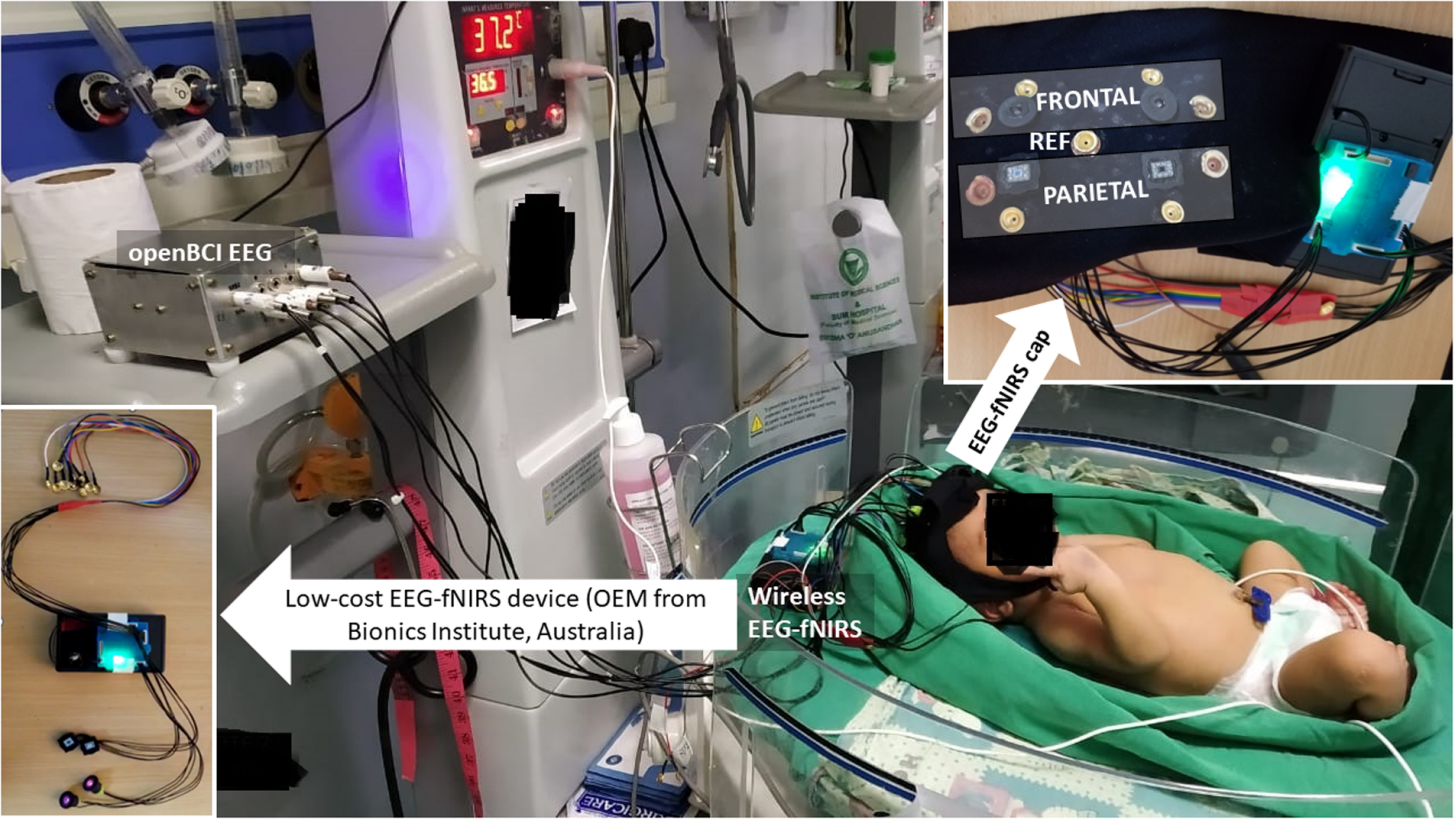
Experimental setup for perinatal human study in the NICU using the low-cost EEG-fNIRS device (OEM from Technische Universität Berlin)—see the bottom left inset. The eight EEG electrodes were distributed bilaterally in the frontal and the parietal areas—see the top right inset. The two fNIRS sources were placed bilaterally in the frontal area, while the two fNIRS detectors were placed bilaterally in the parietal area in the cap (using a black cloth headband). NICU, neonatal intensive care unit; EEG, electroencephalogram; fNIRS, functional near-infrared spectroscopy.

The experimental setup is shown in Figure 3, where the parietal EEG channels were averaged and subtracted from the averaged frontal channels to get a single channel EEG data. The bilateral frontal–parietal fNIRS channels were also averaged to get a single channel of fNIRS data. The preprocessing used in the perinatal ovine model study was applied to the human EEG-fNIRS data. The first-level LDA classifier [trained using human perinatal EEG data from the CHB-MIT dataset (19)—see section *Preprocessing—seizure binary classifier*] was applied to label the seizure segments in the EEG data. Then, the second-level SVM classifier in the hierarchical classifier, trained using the ovine EEG-fNIRS data, was applied to 60-s sliding windows of the human EEG-fNIRS data to label the hypoxia and the control (normal) states. Then, for mechanistic investigation of the NVC system using modal analysis (33), we applied EMA using the ARX system model (“arx”, System Identification Toolbox). Here, we performed EMA of the estimated NVC system that was estimated from the EEG-fNIRS data. Input and output time series were stored using a data object in the time domain (“iddata” in MATLAB). We used the modal analysis functions “modalfrf” to determine the FRFs, “modalfit” to determine the modal parameters of the FRF, and “modalsd” to generate a stabilization diagram for the modal analysis in MATLAB (MathWorks, Inc.). A single set of modal parameters was generated using the least-squares complex exponential (LSCE) algorithm in MATLAB (MathWorks, Inc.) by analyzing multiple response signals simultaneously in “modalsd”. Then, a stabilization diagram was used to identify the physical modes by examining the stability of the poles as the number of modes increased. Here, the given pole was considered stable in frequency if its natural frequency changes by less than 1% and stable in damping if the damping ratio changes by less than 2% as the model order increases in the stabilization diagram.

## 3. Results

### 3.1. Results from the perinatal ovine study—classification based on autoregressive parameters

**Figure 4:**
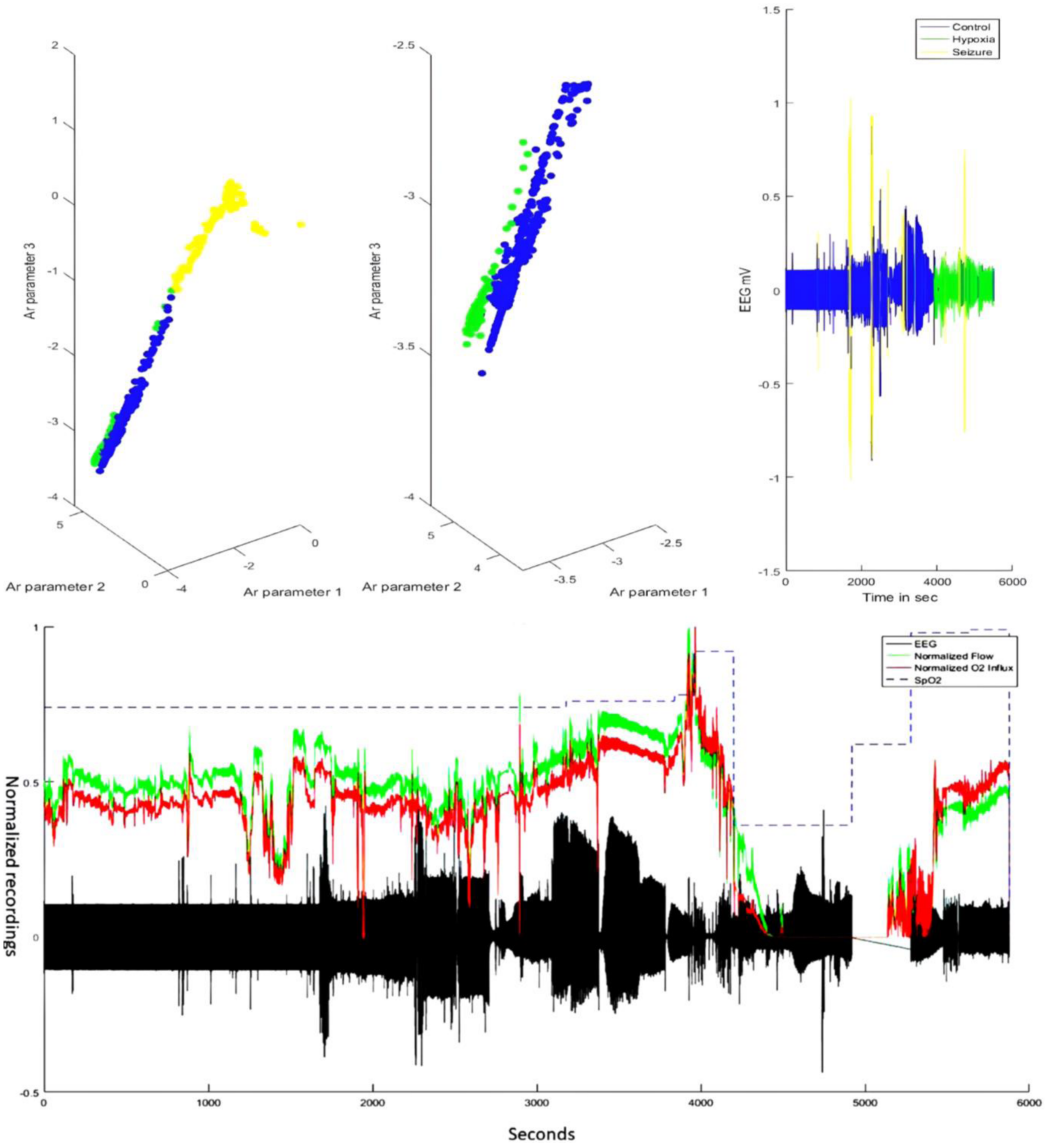
Illustrative example of subject 11/16-1—comparing EEG classifier labels vis-à-vis carotid blood flow and oxygen influx data. The top panel shows the labeling by the hierarchical classifier in the AR feature space that could discriminate the brain state—control or normal (blue), hypoxia (green), and seizure (yellow). The bottom panel shows the corresponding carotid flow and oxygen (O_2_) influx from the oxygen saturation (SpO_2_) data. Note that around the 4,000-s timepoint, the carotid blood flow increase is followed by a steep decrease—the response of the neonate to asphyxial arrest (21). The chest compression and resuscitation data sections were removed (around 5,000 seconds), which was followed by ROSC with the return of the carotid flow and oxygen influx. Here, the gap in the EEG data (in black) before ROSC and after the asphyxial arrest is due to the removal of the artifactual (due to chest compression) EEG data. EEG, electroencephalogram; AR, autoregressive; ROSC, spontaneous return of circulation.

Clustering of the AR parameters for the experimentally induced HIE states in the ovine model (see the upper panel of Figure 4) allowed binary decision planes for the first level (seizure vs. nonseizure EEG data) and the second level (hypoxia vs. control EEG data) in the hierarchical linear classifier using a sliding window of 3.4 s for the EEG data. Here, a sliding window of 3.4 s [longer than 2 s used in the prior work with the SVM classifier (19)] was found to be adequate for the estimation of AR parameters for the reconstruction of the EEG power spectrum (22)—more details are in the thesis (24). The first level of the hierarchical linear classifier determined the seizure state using the LDA classifier applied to EEG data in sliding windows of 3.4 s, where the confusion matrix of the binary classifier trained with the human CHB-MIT dataset is shown in Table 2. Here, the accuracy is 92.68%, sensitivity is 76.88%, and specificity is 93%, which are comparable to those in the prior work (19). Then, the decision plane of the LDA seizure classifier was used to identify and label the seizure data segments (=3.4 s) in the perinatal ovine EEG data. Then, the second-level classifier was trained to separate the control (normal) segment from the hypoxia segment using either the AR parameters from EEG data (with a 3.4-s sliding window) or the ARX parameters from the EEG-fNIRS (with a 60-s sliding window). AR parameters performed moderately well to separate the control (normal) segment from the hypoxia segment (see Table 3), where the accuracy was 98.44%, sensitivity was 70.75%, and specificity was 81.78%. However, with ARX parameters from EEG-fNIRS data, the classifier performance to separate the control (normal) segment from the hypoxia segment (see Table 4) (also Supplementary material Figures S1–S3) improved sensitivity and specificity, with the accuracy at 95.30%, sensitivity at 91.95%, and specificity at 96.75%. Figure 4 shows an illustrative example of the correspondence of the classification of the control (normal) segment from the hypoxia segment vis-à-vis normalized carotid flow recordings and global hypoxia (oxygen influx based on oxygen saturation).

**Table 2:** Confusion matrix of the ictal state classification using the LDA classifier.

*N* = 8,670	Predicted class		
	8,670	*P*	*N*
Actual Class	P	143	43
	N	592	7,892

LDA, linear discriminant classifier.

**Table 3:** Confusion matrix of the hypoxia classification based on AR parameters from EEG using the SVM classifier.

*N* = 8,484	Predicted class		
	8,484	*P*	*N*
Actual class	P	1,819	752
	N	1,077	4,836

EEG, electroencephalogram; AR, autoregressive; SVM, support vector machine.

**Table 4:** Confusion matrix of the hypoxia classification based on ARX parameters from EEG-fNIRS using the SVM classifier.

*N* = 8,484	Predicted class		
	8,484	*P*	*N*
Actual class	P	2,364	207
	N	192	5,721

EEG, electroencephalogram; fNIRS, functional near-infrared spectroscopy.

### 3.2. Results from the human feasibility study

A nontechnical staff was trained to conduct cot-side continuous EEG-fNIRS data acquisition in NICU that was established in a limited resource setting with 1 day of shadowing of a technical expert to learn the experimental protocol—the setup is shown in Figure 3. The two-level hierarchical classifier developed using the ovine EEG-fNIRS data (see Figure 4) was applied to the human EEG-fNIRS data. The hierarchical classifier labeled the six severe HIE cases and four severe HIE cases with sepsis as “hypoxia” and the four moderate HIE cases as the “control”—hypoxia and control labels are based on the perinatal ovine experiment (see Figure 4). Here, EMA provided insights into the NVC modes (33), where the severe HIE and the severe HIE with sepsis cases were found to be different in the stabilization diagram.

### 3.3. Results from the EMA of the human NVC

**Figure 5:**
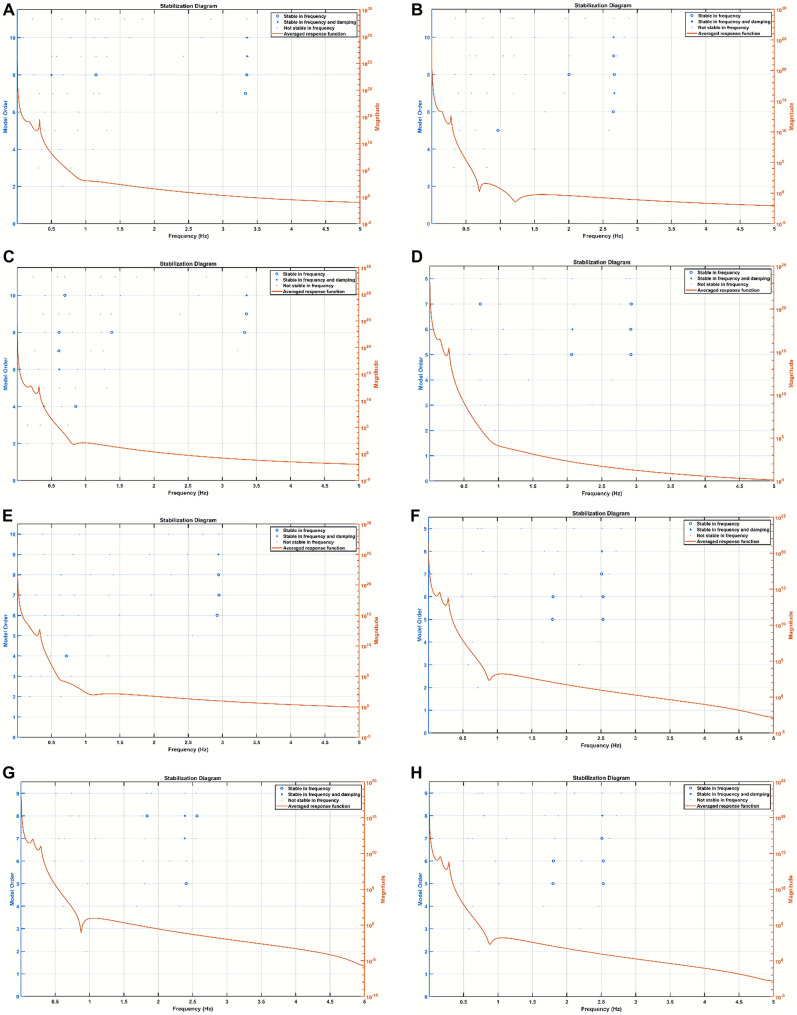

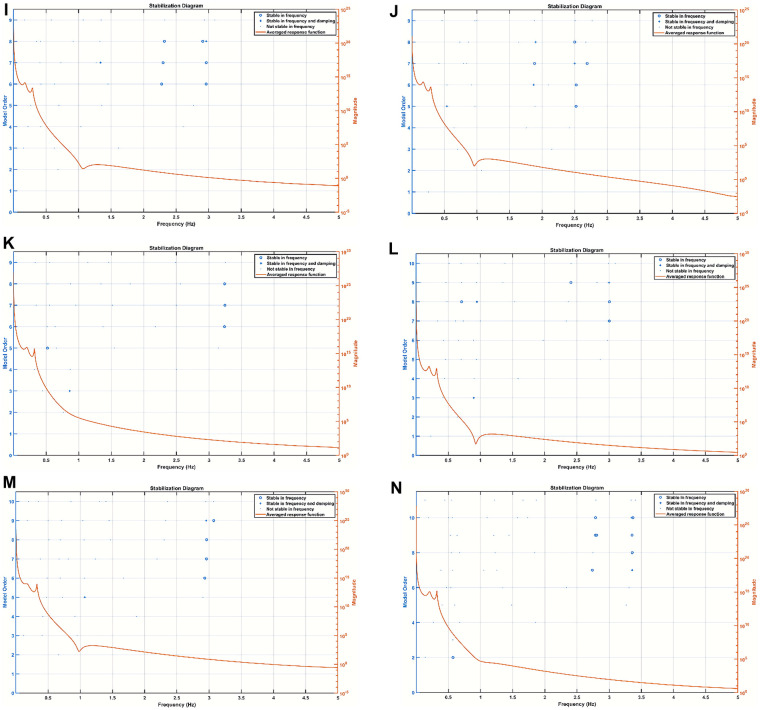
Stabilization diagram of the ARX model of the neurovascular coupling system. (**A**) Subj 1: moderate HIE, (**B**) subj 2: severe HIE with sepsis, (**C**) subj 3: severe HIE with sepsis, (**D**) subj 4: moderate HIE, (**E**) subj 5: severe HIE with sepsis, (**F**) subj 6: severe HIE, (**G**) subj 7: severe HIE, (**H**) subj 8: severe HIE, (**I**) subj 9: severe HIE, (**J**) subj 10: severe HIE, (**K**) subj 11: moderate HIE, (**L**) subj 12: severe HIE with sepsis, (**M**) subj 13: severe HIE, and (**N**) subj 14: moderate HIE. ARX, autoregressive with extra input; HIE, hypoxic-ischemic encephalopathy.

Figure 5 shows the stabilization diagrams of the NVC system estimated from the EEG-fNIRS signals (60-s sliding window) from six severe HIE human perinatal cases, four severe HIE human perinatal cases with sepsis, and four moderate HIE human perinatal cases. Here, the stabilization diagrams of the four moderate HIE human perinatal cases did not show a dip at around 1 Hz in the averaged frequency response function; however, the four severe HIE human perinatal cases with sepsis had a dip at around 1 Hz and a stable pole mainly in the frequency between 0.5 and 1 Hz—is this related to respirocardiac dysfunction?

## 4. Discussion

Our study showed the feasibility of training an SVM classifier with the ARX parameters from the perinatal ovine model EEG-fNIRS data and then applying the SVM classifier to the human data to detect HIE severity. The SVM classifier was at the second level of our hierarchical classifier, where the first level was an LDA seizure classifier that was trained using the human CHB-MIT dataset (18). The LDA seizure classifier had an accuracy of 92.68%, a sensitivity of 76.88%, and a specificity of 93%, which were comparable to those in the published prior work using the CHB-MIT dataset (19). Then, the trained LDA seizure classifier was used to label the seizure data segments (=3.4 s) in the perinatal ovine model EEG data and the human EEG data. ARX parameters from the EEG-fNIRS seizure-free data segments (in 60-s windows) from the perinatal ovine model achieved an accuracy of 95.30%, a sensitivity of 91.95%, and a specificity of 96.75%. The perinatal ovine model-trained hierarchical classifier was applied to the human data, where it labeled the six severe HIE cases and four severe HIE cases with sepsis as “hypoxia” and the four moderate HIE cases as the “control.” Therefore, we showed the technical feasibility of our two-level hierarchical classifier in differentiating severe HIE from moderate HIE, which is feasible for hardware implementation (34).

Prolonged hypoxic events in the ovine model led to substantial seizure activity when the neonates were inherently susceptible to seizures with many more excitatory synapses than inhibitory synapses. So, the seizure data segments (=3.4 s) were removed using the LDA classifier trained with the CHB-MIT dataset (18) before second-level HIE classification. Here, the LDA classifier used only EEG data for the classification of the seizure data segments since the manually labeled CHB-MIT dataset did not provide simultaneous fNIRS data. Then, our second-level SVM classifier using ARX parameters from the EEG-fNIRS seizure-free data segments (in 60 s windows) performed better in terms of sensitivity and specificity than the SVM classifier using AR parameters from the EEG seizure-free data segments—see Tables 3, 4. Indeed, tissue oxygenation and hemodynamics can provide additional information (35) including seizure effects on the neurovascular tissue (36), as shown by our perinatal ovine model data (see that bottom panel of Figure 4), that may guide the hemodynamic care (37), especially in severe HIE cases with seizure load, which is time-critical (38). Under oxygen starvation, an extracellular increase in the gamma-aminobutyric acid (GABA), the most common inhibitory neurotransmitter, can help in metabolic suppression (39), which correlates with the hemodynamics and neurovascular coupling (40)—the excitation/inhibition (E/I) ratio can be estimated with EEG-fNIRS (41). Importantly, the neurodevelopmental circuits in neonates under HIE insults may maladaptively coordinate their excitatory and inhibitory inputs to establish an E/I ratio (42), where neuroenergetics may play a crucial role (43). For example, hypoglycemia may reduce GABA levels due to ATP depletion in the hypoxia state (44). Also, the HIE effects on the cerebellum (45) may be underestimated (46), which needs future development of whole head fNIRS technology (47, 48) for neonates as the thin skin and skull allow deep penetration of the NIR light. Cerebellar Purkinje fibers are sensitive to hypoxic injury and can show damage even in the mild cases of HIE (49). Indeed, HIE accounts for chronic cerebellar deficits, including schizophrenia and other nonaffective psychoses (50), which create an economic burden; hence, low-cost technological innovations are crucial (51).

Our perinatal ovine hypoxia model benefited from previous studies on hypoxia-ischemia animal models for a mechanistic understanding of the SVM classifier results. In the study by Bjorkman et al. (52), the ictal activity was subclassified into two subgroups, clinical and subclinical. Clinical seizures had some visual effect on movement, limb jerks, or mouth quivering, while subclinical seizures can only be detected by abnormalities in the EEG with the absence of movement. In this study on 28 piglets with 77% ictal activity, the background EEG showed lower amplitude compared to that of the nonseizure ischemic state. This supports our SVM classifier approach, where we analyzed the background EEG after removing seizure data segments (using the first-level LDA seizure classifier). Here, a lower amplitude background EEG activity can be a marker of increased neurological damage, where the importance of a lower amplitude background EEG activity was shown by histological analysis after euthanasia (52). Then, fast oscillations (>40 Hz) in neonatal EEG are rare, and high gamma frequencies (27) evaded our first-level LDA classifier. More advanced seizure detection methods are available to identify fast oscillations (>40 Hz) (27); for example, in previous publications (53, 54), researchers have used backpropagation neural networks (53) with an input layer of 9 neurons, a hidden layer of 2–3 neurons, and an output layer containing 1 neuron. The input layer was trained on statistical measures of the ictal waveform itself. Researchers used this classifier to identify the differences between EEG activity and obtained a 93.75% accuracy. Another group (54) attempted to identify all ictal activity with one classifier by dividing peaks that are separated enough to be considered seizures, that is, at least 100 ms. Then, the researchers analyzed the portions of the wave before and after the peak with the amplitude difference and the duration of the wave. These parameters of each half-wave were used to train an SVM with a high sensitivity of 97%. Many advanced machine learning algorithms are under development; however, their clinical utility beyond conventional EEG needs further investigation, especially in limited resource settings (55).

In the current study, we showed the importance of EMA of the NVC system estimated from the seizure-free background EEG and fNIRS data that provided insights. Here, stabilization diagrams with and without stable poles were found for the different cases of severe HIE, severe HIE with sepsis, and moderate HIE. However, the clinical and physiological significance of the dip at around 1 Hz and a stable pole mainly in the frequency between 0.5 and 1 Hz in the four severe HIE human perinatal cases with sepsis (see Figure 5) needs a larger clinical study with another control group with depressed neonates without HIE. Here, changes in the NVC due to HIE have been demonstrated by previous works by Chalak’s group (6, 9); however, our system analysis using EMA may provide further insights into the neurovascular (and neurometabolic) dynamics. Neurovascular (and neurometabolic) dynamics is also relevant to adult acute brain injury cases, where normalization of neurovascular coupling may herald recovery of consciousness (56). Here, the effects of seizure activity on the coupling dynamics of the neural activity (measured with EEG) with the cerebral metabolism, oxygen delivery, and blood volume may be crucial to guide medication (36, 57), especially by leveraging optical monitoring in neonates (58). Other relevant chromophores, cytochrome c oxidase (CCO) and water, can also be investigated with optical monitoring in the neonates (58), which was developed in another study by adding four different wavelengths (780, 810, 820, and 840 nm) to the low-cost EEG-fNIRS sensor (https://neuromodec.org/nyc-neuromodulation-online-2020/P18.html) (59). In that case series (59), we found that neurometabolic coupling was specifically affected in HIE with sepsis, which may be related to the differences in the stabilization diagrams (see Figure 5) between the six severe HIE human perinatal cases and the four severe HIE human perinatal cases with sepsis. Howard et al. (58) highlighted the importance of the estimation of the oxidation state of the CCO (oxCCO) concentration changes in HIE. Here, CCO is essential to generate ATP efficiently during aerobic respiration, so the effects of seizure activity on the background EEG and oxCCO will be important to study its metabolic effects (58). Then, Howard et al. (58) reviewed the literature that showed preictal changes in the cerebral hemodynamics that aligns with our perinatal ovine data (24)—see Figure 4. Figure 4 shows a small increase in the preictal carotid artery flow that was also detected with fNIRS and may improve the latency [or even predict (60)] of ictal period classification when fNIRS is added to EEG monitoring of seizure activity. Also, the accuracy of the ictal period classification may be improved with multimodal EEG-fNIRS data due to the primarily biphasic response of oxyhemoglobin and deoxyhemoglobin concentration changes (58). Nevertheless, the hemodynamic responses to seizures are not uniform across the literature (58), and the individual differences in the neurovascular and neurometabolic coupling may subserve the effects of seizures on the brain tissue (57). For example, any progressive decrease in oxCCO baseline with sequential seizures (61) needs future investigation vis-à-vis clinical outcomes including exacerbation of epileptogenesis following HIE (62).

Hypoglycemia is a common metabolic problem among malnourished newborn babies (63), which can also disturb brain metabolism in HIE. A multiscale model will be needed for the mechanistic understanding of the hypoglycemia effects on the outcome from HIE and sequential seizure events. Sepsis is characterized by systemic changes in the metabolism (64) that can further disturb brain metabolism in HIE where optical monitoring can provide insights (58). Prior work by Jolivet et al. (65) provided a detailed neurometabolic model that captured the concentration of lactate in the neuronal, astrocytic, and extracellular compartments that was coupled as modulatory feedback (66, 67) with the voltage of the neuronal membrane. Such mechanistic investigation is crucial since oxygen and glucose deprivation can lead to an increase in the extracellular concentrations of excitatory amino acid neurotransmitters (68), leading to an E-I imbalance in the brain tissue (at the level of neuronal circuits) (43). Then, neuronal circuits may try to self-organize toward E-I balance (69) via changes in the connectivity that can be dysfunctional when there is a genetic risk (70, 71). Also, hypoxia-ischemia-induced gene transcription effects are possible (72). Previous work on patient-derived cerebral organoids has revealed gene expression patterns suggesting dysregulation of mitochondrial function (73) that can lead to long-term deficits in synaptic E-I balance in susceptible individuals. Such gene–environment interactions can be investigated mechanistically using a subject-specific brain organoid model from human-induced pluripotent stem cells (iPSCs) to test optical theranostics (59). Then, oxygen–glucose deprivation can be implemented in an *in vitro* subject-specific brain organoid model (59) for mechanistic studies. Notably, our *in vitro* subject-specific brain organoid study (https://neuromodec.org/nyc-neuromodulation-online-2020/P18.html) (59) showed an increase in the CCO activity and pH in the organoid tissue and a decrease in the electrophysiological spectral exponent [related to the E-I balance (74)] following photobiomodulation. These preliminary results are important for future works on nonpharmacological therapeutics since histogenous hypoxia and acid retention are closely related to glucose metabolism (71) that may be photobiomodulated (https://neuromodec.org/nyc-neuromodulation-online-2020/P18.html) (59), which needs future investigation. In phase zero studies (43), the brain organoid platform (59) can use a dual-polymer sensor in the Matrigel matrix to provide real-time glucose and oxygen monitoring (75) during mitochondrial photobiomodulation to capture the neurometabolic dose/response relationship for individualized delivery (33). However, our brain organoid platform (59) cannot currently model neurovascular coupling, which may be feasible with vascularized organoids (76).

In conclusion, the current study showed the feasibility of multimodal EEG-fNIRS data acquisition and the EMA approach for the systems analysis of NVC that may provide biomarkers of the sepsis effects on the neurovascular brain tissue in human HIE. Here, the EMA approach to the NVC dynamics using EEG-fNIRS data is novel in our knowledge; however, the systems analysis may need to be extended beyond the neurovascular bundle (77) to include noninvasive measurements of blood pressure and cardiac output (e.g., electrocardiogram of the heart rate) in the human studies [see Figure 1 and the published results from the perinatal asphyxiated lamb model experiments (26)]. Then, cerebral blood flow (CBF) is regulated by cerebral autoregulation, cerebral vasoreactivity, and neurometabolic coupling (78, 79), which can be monitored using cerebral near-infrared spectroscopy (35). Also, seizure-induced autonomic dysfunction is possible (80), which requires systems analysis beyond EEG and fNIRS with the inclusion of simultaneous blood pressure and cardiac monitoring. Here, the effect of the preictal increase in the CBF during a severe metabolic deficit in HIE (e.g., slowing of background EEG) may be physiologically important (52, 81–83)—see Supplementary material Figure S4 from the perinatal asphyxiated lamb model experiments in the Supplementary Material. So, a unified theory of seizure-induced brain state abnormalities including the effects of sepsis in HIE, which may share a common point of origin with hypoperfusion/hypoxia (57), needs future investigation for the development of a robust biomarker amenable to optical brain tissue monitoring in the neonates (58).

## Data Availability

The original contributions presented in the study are included in the article/**Supplementary Material**, further inquiries can be directed to the corresponding author.

## Supplementary material

Supplementary Figure S1

AR parameter 1 in the three cases: control, hypoxic, and ictal states. The control and hypoxic clusters have average separation but are not completely separated, while the ictal activity is clearly separated.

Supplementary Figure S2

AR parameter 2 in the three cases: control, hypoxic, and ictal states. The control and hypoxic clusters have average separation but are not completely separated, while the ictal activity is clearly separated.

Supplementary Figure S3

AR parameter 3 in the three cases: control, hypoxic, and ictal states. The control and hypoxic clusters have average separation but are not completely separated, while the ictal activity is clearly separated.

Supplementary Figure S4

Reduction in the amplitude of the discharges in EEG (black line) during an increase in the normalized flow (green line) and normalized oxygen (O_2_, red line) delivery (based on constant oxygen saturation, SpO_2_, dashed black line).
