# Supplementary material for: Technical feasibility of multimodal imaging in neonatal hypoxic-ischemic encephalopathy from an ovine model to a human case series
Source: Front Pediatr. 2023 Jun 22;11:1072663. doi: 10.3389/fped.2023.1072663 (PMC10323750; doi:10.3389/fped.2023.1072663)
Supplement: Supplementary file 1 [file Datasheet1.pdf]

Supplementary Material

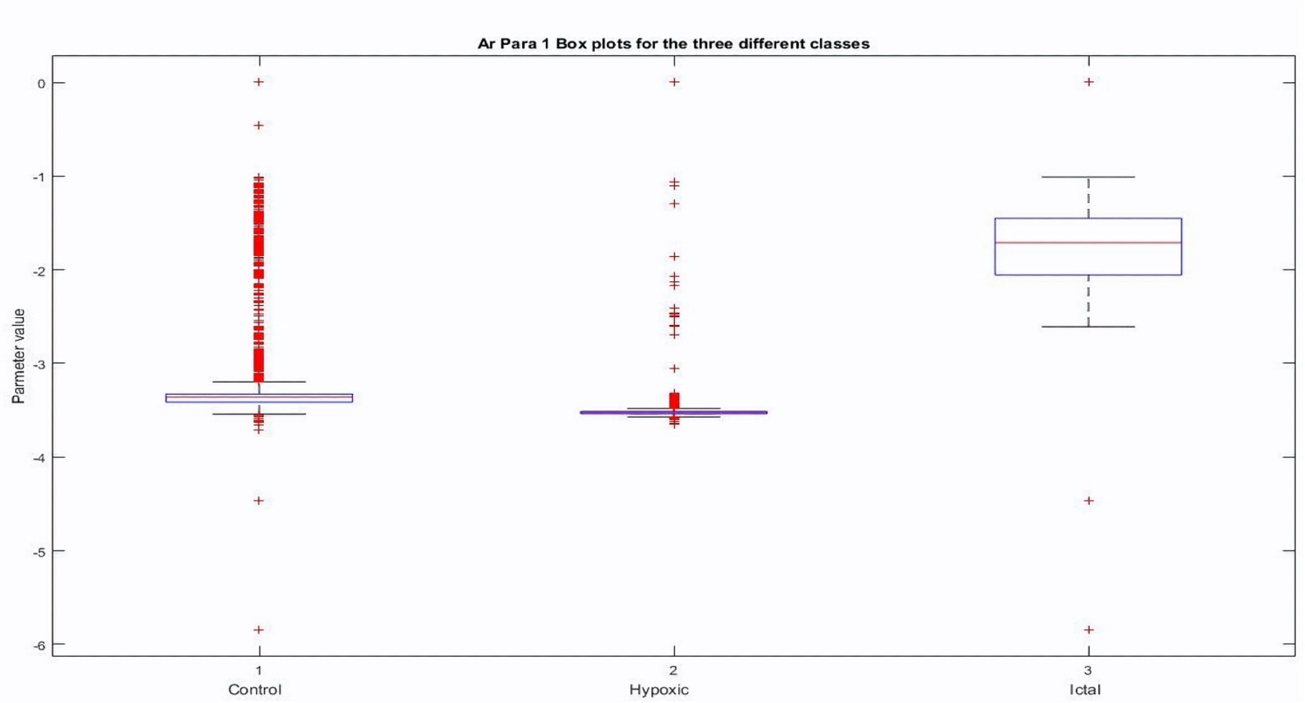

Figure S1: AR parameter 1 in the three cases, Control, Hypoxic, and Ictal states. The Control and Hypoxic clusters have average separation but are not completely separated while the ictal activity is clearly separated.

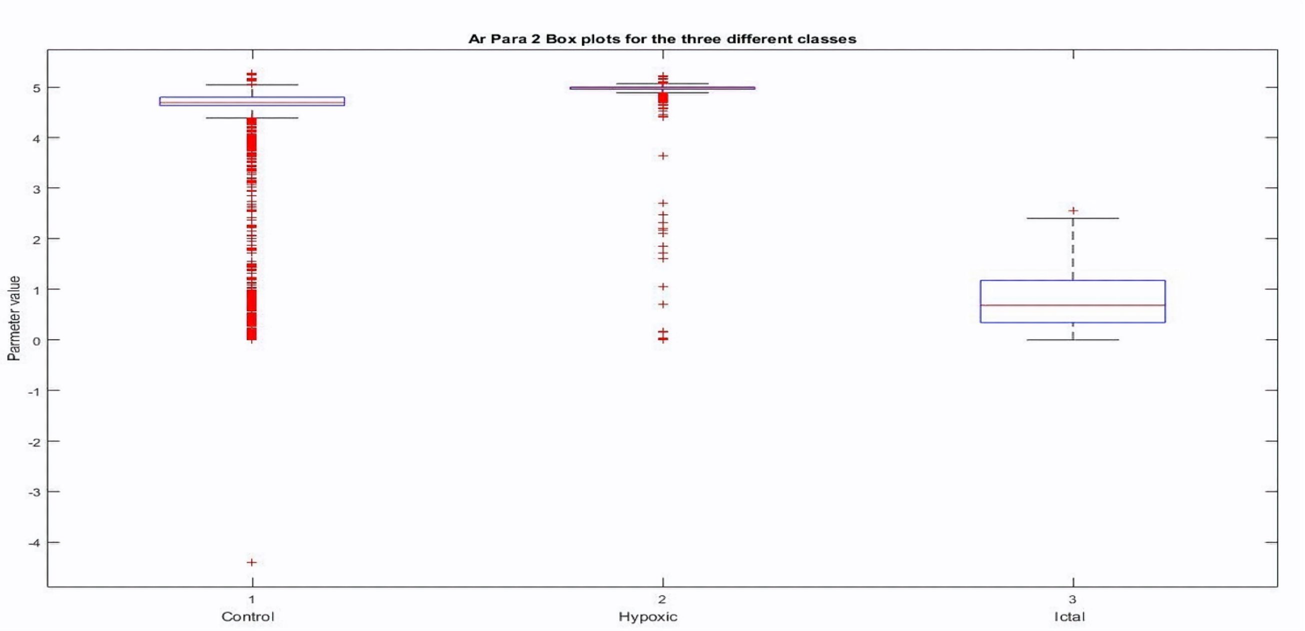

Figure S2: AR parameter 2 in the three cases, Control, Hypoxic, and Ictal states. The Control and Hypoxic clusters have average separation but are not completely separated while the ictal activity is clearly separated.

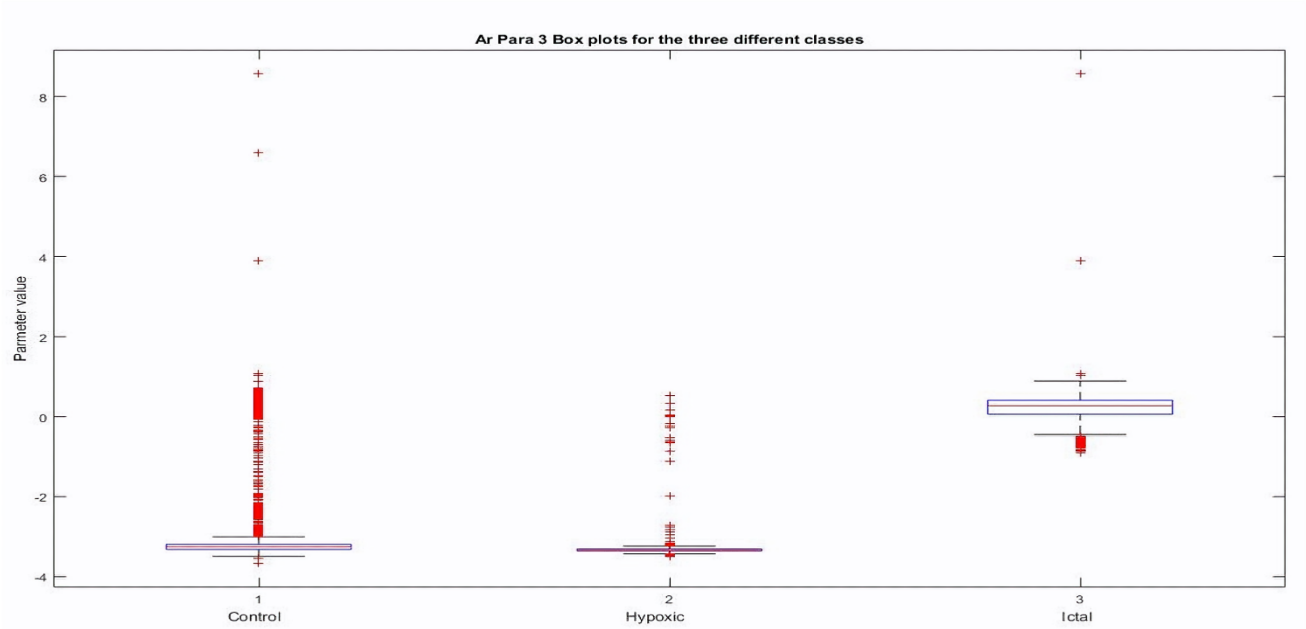

Figure S3: AR parameter 3 in the three cases, Control, Hypoxic, and Ictal states. The Control and Hypoxic clusters have average separation but are not completely separated while the ictal activity is clearly separated.

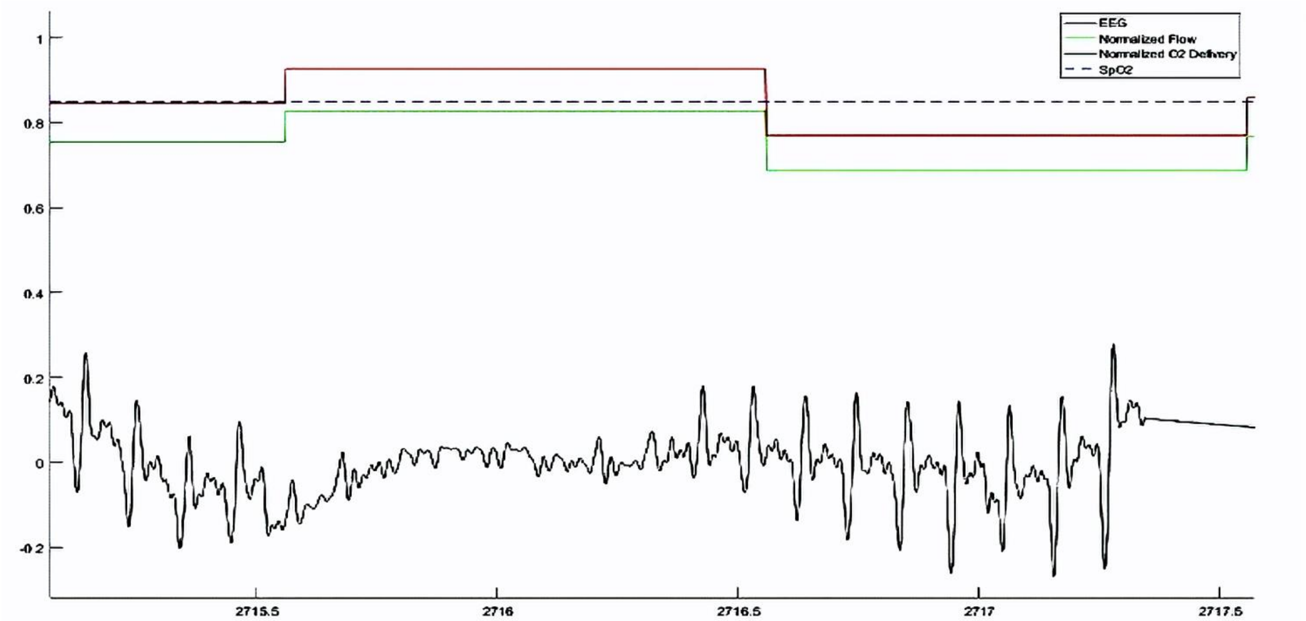

Figure S4: A reduction in the amplitude of the discharges in EEG (black line) during an increase in the normalized flow (green line) and normalized oxygen (O2, red line) delivery (based on constant oxygen saturation, SpO2, dash black line).
